# Acidic microenvironment-induced LAMC2 expression promotes proliferation, migration, and pathway regulation in non-small cell lung cancer cells

**DOI:** 10.3389/fonc.2026.1747868

**Published:** 2026-03-24

**Authors:** Lu Wang, Dengliang Huang, Yaogang Zhang, Jing Hou, Meiyuan Tian, Jun Jiang, Yanyan Ma

**Affiliations:** 1 Research Center for High Altitude Medicine, Key Laboratory of High-Altitude Medicine (Ministry of Education), Key Laboratory of Application and Foundation for High Altitude Medicine Research in Qinghai Province (Qinghai-Utah Joint Research Key Lab for High Altitude Medicine), Qinghai University, Xining, China; 2 Department of Hematology, Qinghai University Affiliated Hospital, Xining, Qinghai, China; 3 Central Laboratory/Key Laboratory of Echinococcosis Research, Qinghai University Affiliated Hospital, Xining, Qinghai, China; 4 Department of Oncology, Qinghai University Affiliated Hospital, Xining, Qinghai, China; 5 Division of Scientific Research Management, Qinghai University Affiliated Hospital, Xining, Qinghai, China

**Keywords:** acidic microenvironment, LAMC2, non-small cell lung cancer, prognosis, tumor progression

## Abstract

**Objective:**

This study aims to investigate the regulatory mechanism by which the acidic microenvironment modulates LAMC2 expression in non-small cell lung cancer (NSCLC) and to elucidate its roles in tumor cell proliferation, migration, invasion, and associated signaling pathways, thereby providing a theoretical foundation for targeted lung cancer therapies.

**Methods:**

A cohort of 104 pathologically confirmed NSCLC patients treated at a designated hospital from January 2018 to December 2021 was enrolled. RNA-seq data from the TCGA database were analyzed to evaluate LAMC2 expression differences and prognostic implications using the GEPIA2 tool. *In vitro* assays assessed the acidic microenvironment’s impact on LAMC2 expression, with functional evaluations of LAMC2 overexpression and knockdown on cellular behaviors.

**Results:**

The LAMC2 mRNA levels were elevated across multiple tumor types and correlated with unfavorable prognoses in lung adenocarcinoma (LUAD) and lung squamous cell carcinoma (LUSC). The acidic microenvironment significantly upregulated LAMC2 expression through HIF-1α-mediated transcriptional activation. LAMC2, in turn, promoted NSCLC cell proliferation, migration, and invasion by activating the PI3K/Akt signaling pathway and inducing epithelial–mesenchymal transition (EMT). Clinically, a high LAMC2 expression was associated with poor tumor differentiation, advanced TNM staging, and reduced 3-year survival rates. Moreover, LAMC2 positively correlated with immune checkpoint molecules (e.g., PD-L1, CTLA-4), implying its involvement in immune evasion.

**Conclusion:**

The acidic microenvironment upregulates LAMC2 via HIF-1α-mediated transcriptional regulation and lactate accumulation, thereby driving malignant progression in NSCLC. Elevated LAMC2 levels are linked to enhanced proliferation, migration, invasion, and dismal patient outcomes, underscoring its potential as a therapeutic target for precision oncology.

## Introduction

Non-small cell lung cancer (NSCLC) represents the predominant subtype of lung cancer, comprising approximately 85% of all cases (1). Amid escalating global air pollution, pervasive smoking, and other environmental risk factors, the NSCLC incidence has surged annually, establishing it as a primary contributor to cancer-related mortality worldwide (2, 3). Although recent advancements in therapeutic modalities—encompassing surgery, radiotherapy, chemotherapy, and immunotherapy—have enhanced the outcomes, most NSCLC diagnoses occur at advanced stages, where current interventions exhibit substantial limitations. Consequently, early detection and precision-targeted therapies persist as formidable hurdles in NSCLC management (4, 5). Thus, the identification of novel therapeutic targets, especially those amenable to early intervention, remains a pivotal imperative in NSCLC research.

Tumorigenesis and progression transcend intrinsic mutations within neoplastic cells, profoundly influenced by the tumor microenvironment (TME), which exerts indispensable regulatory effects (6, 7). The TME encompasses tumor cells alongside infiltrating immune cells, vasculature, fibroblasts, and diverse extracellular matrix (ECM) constituents (8). It furnishes a nurturing scaffold that sustains tumor proliferation, dissemination, and metastatic dissemination. Within this milieu, the acidic microenvironment emerges as a salient modulator (9). Tumor hypoxia and nutrient scarcity during rapid proliferation precipitate lactate accumulation, engendering a locally acidic milieu (10). This acidity not only reprograms tumor cell metabolism but also profoundly modulates their oncogenic traits, including enhanced invasiveness and motility (11). Evidence indicates that acidic conditions remodel ECM architecture and composition, thereby fostering epithelial–mesenchymal transition (EMT) in tumor cells and amplifying invasive and metastatic propensities (12). Elucidating the interplay between the acidic TME and tumor cell biology, particularly in NSCLC, holds profound implications for delineating oncogenic mechanisms and devising innovative therapeutic paradigms.

LAMC2 (laminin subunit gamma 2), an integral ECM glycoprotein of the laminin family, orchestrates critical cellular processes (13). Laminins, as key ECM scaffolds, underpin cell polarity, adhesion, migration, and differentiation (14). As the γ subunit, LAMC2 assembles with α and β chains to form functional laminin heterotrimers, predominantly localized to basement membranes under physiological states to mediate cell–matrix interactions. In oncogenesis, LAMC2 expression is aberrantly upregulated across diverse malignancies, correlating with augmented tumor cell proliferation, invasion, and metastasis (15). Beyond structural contributions to the ECM, LAMC2 engages membrane receptors to activate downstream signaling cascades, thereby dictating tumor cell phenotypes (16). Elevated LAMC2 levels have been documented in entities such as breast and hepatocellular carcinomas, where they portend heightened invasiveness and metastatic risk (17, 18). Previous studies have implicated LAMC2 in NSCLC progression, highlighting its overexpression as a driver of tumor invasion and metastasis. However, the regulatory effects of the acidic TME on LAMC2 expression and function in NSCLC remain largely unexplored, representing a critical knowledge gap.

This investigation aims to delineate the regulatory dynamics of the acidic microenvironment on LAMC2 expression while illuminating its latent functionalities within the NSCLC TME. By interrogating LAMC2’s conduct in this prototypical lung malignancy under acidic conditions, our study furnishes novel mechanistic insights and directional cues to advance tumor immunotherapy and precision oncology. Unveiling LAMC2’s precise contributions to NSCLC progression may unearth viable therapeutic vulnerabilities, paving the way for LAMC2-targeted interventions that ameliorate patient prognoses.

## Methods and materials

### Clinical sample sources and inclusion/exclusion criteria

This study included 104 patients diagnosed with non-small cell lung cancer (NSCLC) by pathology at a hospital from January 2018 to December 2021. The inclusion criteria were as follows: diagnosis of primary NSCLC by pathology, histological subtypes of adenocarcinoma or squamous carcinoma; age ≥18 and ≤80 years; newly diagnosed patients; surgical treatment with available cancer tissue and matched adjacent normal tissue (≥5 cm from the tumor edge); clinical stages I–IV (AJCC 8th edition). The exclusion criteria were as follows: co-existing other malignant tumors; loss to follow-up or incomplete clinical data; history of severe heart disease (such as severe heart failure or myocardial infarction) that might affect treatment interventions; inability to assess the condition (such as loss of consciousness or inability to cooperate with diagnosis and treatment). This study was approved by the hospital’s Ethics Committee.

### TCGA data selection criteria

This study utilized RNA-seq data from 33 tumor types in the TCGA database (https://portal.gdc.cancer.gov/) (19) and analyzed the expression differences of LAMC2 using the GEPIA2 tool (http://gepia2.cancer-pku.cn/) (20). The comparison between cancerous and adjacent tissues used a significance threshold of |log_2_FC| >1 and *p* < 0.05. Survival analysis was based on the surv_cutpoint function from the survival package to calculate the optimal cutoff value for LAMC2 mRNA expression (FPKM). This analysis yielded an optimal cutoff of 7.18 (log2[FPKM + 1]), which was used to stratify TCGA patients into high- and low-expression groups for survival and correlation analyses. Prognostic differences were evaluated using the log-rank test. The correlation between immune checkpoint molecules (CD274, CTLA-4, etc.) and LAMC2 expression was calculated using Pearson or Spearman correlation analysis, with significance set at *p* < 0.05 and |*r*| >0.1.

### Cell lines and culture conditions

Human normal lung epithelial BEAS-2B cells (ATCC^®^ CRL-9609™) and lung cancer cell lines A549 (Procell, CL-0016, lung adenocarcinoma) and HCC827 (Procell, CL-0094, lung adenocarcinoma) were obtained from the indicated suppliers. The cells were cultured in RPMI-1640 medium (Gibco, 11875093) supplemented with 10% fetal bovine serum (Biological Industries, 10270106) at 37°C in a humidified incubator with 5% CO_2_. The cells were subcultured every 2 to 3 days using 0.25% trypsin (Gibco, 25200056), and only cells with viability greater than 95% were used for the experiments. No antibiotics were added during cell culture.

### Acidic microenvironment treatment

This study analyzed the regulatory effects of the acidic microenvironment on LAMC2 expression using multiple experimental groups. Initially, HCC827 and A549 cells were treated with varying concentrations of lactate (0, 10, 20, and 30 mM; Sigma-Aldrich, USA) for 24 h to observe the concentration-dependent changes in LAMC2 expression. Subsequently, the 20-mM-lactate-treatment group was selected, and LAMC2 dynamic expression was assessed at 0-, 6-, 12-, and 24-h time points. To comprehensively evaluate the impact of different acidic microenvironments, four treatment conditions were set: normal control (normoxia, pH 7.4, no lactate), hydrochloric acid acidosis (pH 6.6), hypoxia (1% O_2_), and lactate treatment (20 mM). All treatments lasted for 24 h, and LAMC2 expression was measured by using qRT-PCR and Western blot. The culture conditions were strictly controlled, with the pH value calibrated using a pH meter and a three-gas incubator maintaining hypoxic conditions to ensure accuracy and reproducibility.

### Cell transfection

A stable overexpression of LAMC2 (LAMC2-OE) and HIF-1α (HIF-1α-OE) was achieved using lentiviral vectors. The GV492 vector, with the element sequence Ubc-MCS-3FLAG-CBh-gcGFP-IRES-puromycin and BamHI/AgeI cloning sites, was used to construct LAMC2-OE and HIF-1α-OE lentiviruses. A negative control lentivirus (NA-OE) was included to ensure specificity. Cells in the logarithmic growth phase were transduced with LAMC2-OE, HIF-1α-OE, or NA-OE lentiviruses in the presence of 5–8 μg/mL Polybrene to enhance transduction efficiency. Following transduction, the cells were incubated at 37 °C with 5% CO_2_ for 48 h to allow a stable gene expression. Cells were then harvested for quantitative real-time PCR (qRT-PCR) and Western blot analysis to evaluate LAMC2 and HIF-1α mRNA and protein expression levels, respectively.

### siRNA knockdown

To achieve the transient knockdown of LAMC2 and HIF-1α, small interfering RNA (siRNA) duplexes were designed and synthesized by TransGen Biotech (Beijing, China). The target sequences were as follows: siLAMC2 (sense: 5′-GGAUCAAGUACUUGAAGAA-3′, antisense: 5′-UUCUUCAAGUACUUGAUCC-3′); siHIF-1α (sense: 5′-CCAUCAACUUCAAGUGAUU-3′, antisense: 5′-AAUCACUUGAAGUUGAUGG-3′); and a non-targeting scramble siRNA (siNC) as negative control. HCC827 and A549 cells in the logarithmic growth phase were seeded in six-well plates (2 × 10^5^ cells/well) and transfected with 50 nM siRNA using Lipofectamine 3000 (Invitrogen, USA) according to the manufacturer’s protocol. Transfection efficiency was assessed 48 h post-transfection by qRT-PCR and Western blot, confirming >70% knockdown. The knockdown cells were then subjected to functional assays (e.g., proliferation, migration) and molecular analyses (e.g., pathway activation) under normoxic or acidic conditions (20 mM lactate). All siRNA experiments were performed in triplicate and repeated independently at least three times to ensure reproducibility.

### Immunohistochemistry

Non-small cell lung cancer tissues and matched adjacent normal tissues (≥5 cm from the tumor edge) were fixed with 10% neutral formalin for 48 h and then embedded in paraffin after dehydration. The paraffin sections were baked at 60 °C for 2 h, deparaffinized with xylene (three times, 10 min each), and rehydrated with graded ethanol (100%, 95%, 80%, and 70%). The slides were incubated with 3% H_2_O_2_ at room temperature for 20 min to block endogenous peroxidase activity. Antigen retrieval was performed using citrate buffer (pH 6.0) in a pressure cooker (121 °C, 2 min), followed by cooling and blocking with 10% normal goat serum at room temperature for 30 min. Sections were incubated overnight at 4 °C with rabbit anti-human LAMC2 antibody (Abcam, ab210959, 1:300) and rabbit anti-human Ki67 (Proteintech, 27309-1-AP, 1:2,000). After washing, the sections were incubated with HRP-conjugated goat anti-rabbit secondary antibody (Proteintech, PR30012, 1:200) for 30 min at room temperature, followed by DAB staining (ZSGB-BIO, ZLI-9018), hematoxylin counterstaining, dehydration, and mounting with neutral resin. Immunoreactivity scores (IS) were calculated by multiplying the staining intensity (0: negative; 1: weak; 2: moderate; 3: strong) by the percentage of positive cells (0: <5%; 1: 5%–25%; 2: 26%–50%; 3: 51%–75%; 4: >75%). The samples were scored independently by two pathologists in a double-blind manner, and disagreements were resolved by a senior pathologist. Adjacent normal lung tissue served as a control for antibody specificity.

### qRT-PCR

HCC827 and A549 cells in the logarithmic growth phase were collected and washed with pre-chilled PBS before RNA extraction using TRIzol reagent (Invitrogen, USA). Clinical NSCLC cancer tissues and matched adjacent normal tissues (≥5 cm from the tumor margin) were frozen in liquid nitrogen, ground into powder, and homogenized in TRIzol. RNA concentration and purity were determined using a NanoDrop spectrophotometer (A260/A280 ratio between 1.8 and 2.1), and RNA integrity was verified by 1% agarose gel electrophoresis, where intact 28S and 18S rRNA bands were clearly observed. For reverse transcription, 1.5 μg of RNA in a final volume of 6 μL (RNase-free water) was mixed with 1 μL of Anchored Oligo(dT)18 primer and 1 μL of Random Primer (TransGen Biotech, Beijing, China). The mixture was incubated at 65 °C for 5 min in a metal bath and immediately chilled on ice for 2 min. Subsequently, 10 μL of 2×ES Reaction Mix, 1 μL of gDNA Remover, and 1 μL of EasyScript RT/RI Enzyme Mix (TransGen Biotech) were added. After brief centrifugation and mixing, reverse transcription was carried out following the manufacturer’s recommended protocol. qPCR was performed using LightCycler 480 Real-Time PCR System (Roche, Switzerland) with SYBR Green Premix. The reaction conditions were as follows: 95°C for 10 min, followed by 45 cycles of 95 °C for 15 s, 60 °C for 34 s, and 50 °C for 30 s. The primer sequences were as follows: LAMC2 (F: 5′-GGAATTTGGACAAGTGCTGTTG-3′, R: 5′-TGACTGGTTGCACTCTGTTCTG-3′), HIF-1α (F: 5′-AGAAACCTATGACCTGCT-3′, R: 5′-AAGCATCCTGTACTGTCCTGTG-3′), and GAPDH (F: 5′-ACAACTTTGGTATCGTGGAAGG-3′, R: 5′-GCCATCACGCCACAGTTTC-3′). A melt curve analysis was performed to confirm amplification specificity. Relative LAMC2 mRNA expression levels were calculated using the 2^−ΔΔCt^ method, with GAPDH as the internal control. Each experimental group was analyzed with three technical replicates, and experiments were independently repeated at least three times. A total of 104 paired clinical tissue samples (tumor and adjacent normal tissues) were included in the analysis.

### Western blot

Cells were washed with pre-chilled PBS and lysed in RIPA buffer containing protease and phosphatase inhibitors for 30 min on ice. After centrifugation at 4 °C, the supernatant was collected, and protein concentration was determined by using the BCA method and adjusted to 2 μg/μL. Then, 30 μg of protein samples was separated by SDS-PAGE, transferred to PVDF membranes, and blocked with 5% skim milk for 1 h. The primary antibodies used were E-cadherin (1:1,000), N-cadherin (1:1,000), p-Akt (Ser473, 1:1,000), p-PI3K (Tyr458, 1:1,000), total Akt (1:1,000), total PI3K (1:1,000), LAMC2 (1:1,000), HIF-1α (1:1,000), c-Myc (1:1,000), and GAPDH (1:5,000), incubated overnight at 4 °C. After washing with PBST, the membranes were incubated with HRP-conjugated secondary antibody (1:5,000) for 1 h at room temperature. Image Lab software was used to analyze the grayscale values.

### Chromatin immunoprecipitation

Cells were crosslinked with 1% formaldehyde at room temperature for 10 min, and chromatin was sheared into 200–500-bp fragments using Bioruptor UCD-200. The chromatin fragments were incubated with HIF-1α antibody (CST, #36169) or IgG control (CST, #2729) at 4 °C overnight, followed by incubation with Protein A/G magnetic beads (Millipore, 16-663) for 4 h. After washing with low salt, high salt, LiCl, and TE buffer, the complexes were crosslinked at 65 °C for 6 h, and DNA was extracted using phenol/chloroform. Specific primers for the HIF-1α binding site in the LAMC2 promoter region (GAACGTGG, core sequence ACGTG) were designed and used for qPCR amplification. The enrichment fold was calculated using the % input method.

### Dual-Luciferase reporter assay

A 2.0-kb upstream fragment of the LAMC2 promoter (−2,000 bp to +100 bp) was cloned into the pGL3-Basic vector (Promega, USA) to generate the pGL3-LAMC2-WT construct, and a mutant reporter (pGL3-LAMC2-MUT) with a disrupted HIF-1α binding site (ACGTG→AAAAA) was created by site-directed mutagenesis. The HCC827 and A549 cells were seeded in 24-well plates (1 × 10^5^ cells/well) and co-transfected with pGL3-LAMC2-WT or pGL3-LAMC2-MUT (400 ng), pcDNA3.1-HIF-1α (200 ng) or empty vector, and pRL-TK Renilla plasmid (20 ng) using Lipofectamine 3000 (Invitrogen, USA). After 36 h of transfection, the cells were exposed to normoxic (pH 7.4) or acidic (20 mM lactate, pH 6.6) conditions for 12 h. Luciferase activity was measured using Dual-Luciferase Reporter Assay System (Promega) and expressed as the Firefly/Renilla ratio normalized to the control. All experiments were performed in triplicate and repeated independently three times.

### Immunofluorescence

Cells were fixed with 4% paraformaldehyde for 15 min and permeabilized with 0.1% Triton X-100 for 10 min. Blocking was done with 5% BSA for 1 h, followed by incubation with primary antibodies against HIF-1α (1:200) and LAMC2 (1:200) at 4 °C overnight. Secondary antibodies Alexa Fluor 488-goat anti-rabbit (1:500) and Cy3-goat anti-mouse (1:500) were incubated at room temperature for 1 h, and nuclei were stained with DAPI (Sigma, D9542). Confocal microscopy (Zeiss LSM 880) was used to observe and analyze the colocalization coefficient (Pearson’s *r*) with ImageJ.

### CCK-8 proliferation assay

Cells were seeded in 96-well plates (3 × 10³ cells/well), and CCK-8 reagent (Solarbio, China) was added at 0, 24, and 48 h. Absorbance at 450 nm was measured using Cytation 5 Cell Imaging Multi-Mode Reader (BioTek, USA). The proliferation rate was calculated as (OD of the experimental group/OD of the control group) × 100%.

### Wound healing assay

Cells were seeded in six-well plates until 90% confluence, and a vertical scratch was made using a 200-μL pipette tip. After washing with PBS, images were captured at 0 and 24 h using an inverted microscope (CKX41 equipped with DP26 camera, Carl Zeiss AG, Germany), and the wound area was measured using ImageJ. Migration rate was calculated as (1 – 24 h area/0 h area) × 100%.

### Transwell invasion assay

Transwell chambers (Corning, 3422) were precoated with Matrigel (1:8 dilution), and 2 × 10^4^ cells were seeded in serum-free medium in the upper chamber. The lower chamber contained 20% FBS. After 24 h, the cells were fixed with 4% paraformaldehyde and stained with 0.1% crystal violet (Solarbio, China). Five random fields were selected to count the invaded cells.

### Patient follow-up

Patients were followed up every 3 months by telephone or outpatient visits, recording survival status until June 2024 or patient death. Overall survival (OS) was defined as the time from surgery to death or the last follow-up.

### Gene set enrichment analysis

Based on RNA-seq data from LUAD and LUSC patients in the TCGA database, R language (v4.2.0) with the survminer package was used to determine the optimal cutoff value for LAMC2 expression (cutoff = 7.18), dividing the patients into high- and low-expression groups. The expression matrix was log2(FPKM + 1)-normalized, and differential genes between the high- and low-expression groups were calculated using the limma package (|log2FC| > 0.5, *p* < 0.05). The pre-ranked gene list was analyzed using the clusterProfiler package (v4.6.2), with reference to the MSigDB database (C2: Reactome pathways, HALLMARK: cancer hallmark gene sets) for pathway enrichment. Key pathways (e.g., glycolysis, epithelial–mesenchymal transition) were visualized with the enrichplot package, showing the enrichment plot and leading-edge genes.

### Statistical analysis

Statistical analysis was performed using SPSS 27.00, R 4.3.3, and GraphPad Prism 10 software. First, data were tested for normality using the Kolmogorov–Smirnov test. For normally distributed data, independent-samples *t*-test was used, with results expressed as mean ± standard deviation (mean ± SD); for non-normally distributed data, rank sum test was performed. Categorical data were expressed as percentages (%) and compared using chi-square test. For multi-time point data, repeated-measures ANOVA (RM-ANOVA) was used with Bonferroni *post-hoc* correction. For comparisons among multiple groups, two-way ANOVA was applied. Correlation analysis was performed using Spearman’s test. Survival analysis was conducted using Kaplan–Meier curves and Cox regression models to assess the impact of related factors on survival. All statistical tests were considered significant at a two-tailed *p*-value <0.05.

## Results

### LAMC2 expression in various tumor types and its impact on lung cancer prognosis

**Figure 1:**
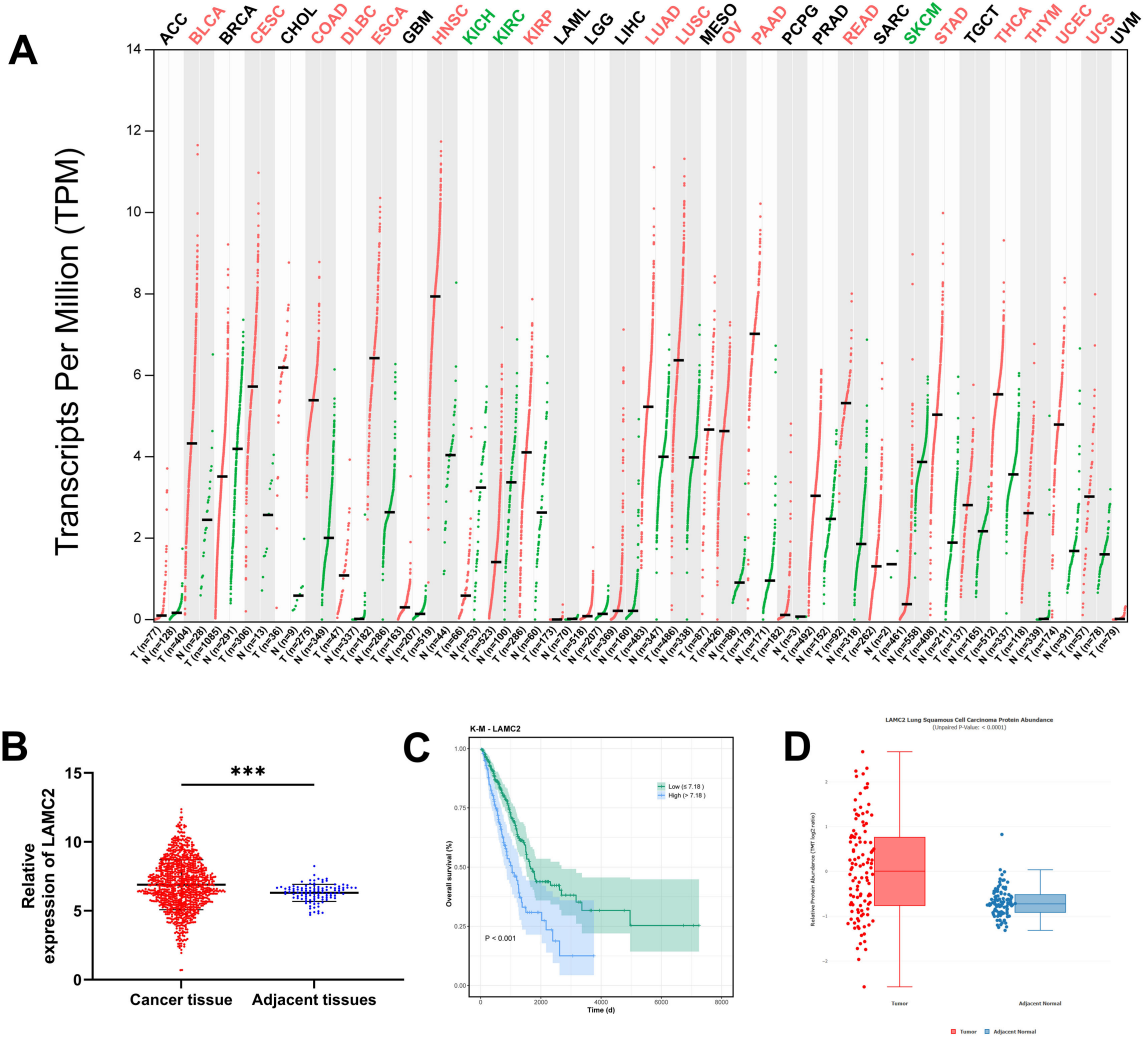
LAMC2 expression in different tumor types and survival analysis in lung cancer. **(A)** LAMC2 expression across different tumor types in TCGA. Tumor types with high expression are marked in red, and those with low expression are marked in green. **(B)** Relative expression differences of LAMC2 between LUAD and LUSC cancer tissues and adjacent normal tissues. **(C)** Survival analysis of LAMC2 in LUAD and LUSC patients, with a cutoff value of 7.18. **(D)** LAMC2 protein expression in LUAD using the cProSite online proteogenomic tool. LAMC2, laminin subunit gamma 2; BLCA, bladder cancer; ESCA, esophageal squamous cell carcinoma; COAD, colorectal cancer; ESCC, esophageal cancer; HNSC, head and neck squamous cell carcinoma; KIRP, kidney cancer; LUAD, lung adenocarcinoma; LUSC, lung squamous cell carcinoma; OV, ovarian cancer; PAAD, pancreatic cancer; READ, rectal cancer; STAD, stomach cancer; THCA, thyroid cancer; THYM, thymic cancer; UCEC, endometrial cancer; UCS, uterine sarcoma. Tumor types with low expression include kidney clear cell carcinoma (KICH), kidney renal clear cell carcinoma (KIRC), and melanoma (SKCM).

Analysis using the GEPIA2 tool from the TCGA database revealed that LAMC2 is highly expressed in several types of cancer, including bladder cancer (BLCA), esophageal squamous cell carcinoma (ESCA), colorectal cancer (COAD), esophageal cancer (ESCC), head and neck squamous cell carcinoma (HNSC), kidney cancer (KIRP), lung adenocarcinoma (LUAD), lung squamous carcinoma (LUSC), ovarian cancer (OV), pancreatic cancer (PAAD), rectal cancer (READ), gastric cancer (STAD), thyroid cancer (THCA), thymic carcinoma (THYM), endometrial carcinoma (UCEC), and uterine sarcoma (UCS). Conversely, it is lowly expressed in kidney clear cell carcinoma (KICH), kidney renal clear cell carcinoma (KIRC), and melanoma (SKCM) (**Figure 1A**). In the comparisons of LUAD and LUSC cancer tissues with adjacent normal tissues, LAMC2 expression was significantly increased (**Figure 1B**), and high LAMC2 expression was associated with poorer survival prognosis in these patients (**Figure 1C**), supporting its potential prognostic role in lung cancer. To further strengthen these findings at the protein level, we analyzed LAMC2 protein expression in LUAD using the cProSite online proteogenomic tool. Consistently, the protein abundance of LAMC2 was significantly elevated in LUAD tumors compared to adjacent normal tissues (*P* < 0.001, **Figure 1D**).

### Correlation between LAMC2 expression and immune checkpoint molecules

**Figure 2:**
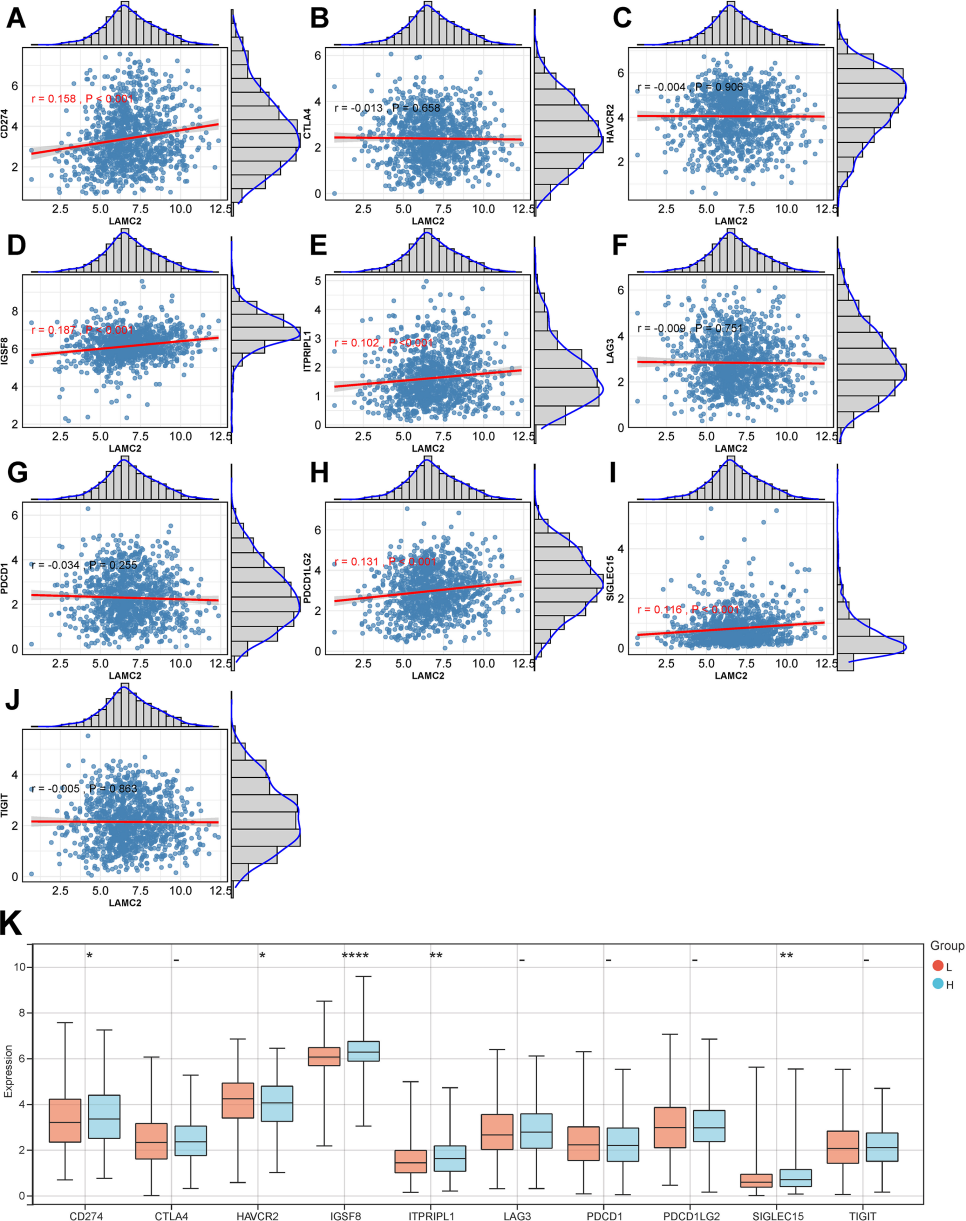
Correlation between LAMC2 mRNA expression and immune checkpoint molecule expression. **(A–J)** Scatter plots showing the correlation between LAMC2 mRNA levels and different immune checkpoint molecules (such as CD274, CTLA-4, HAVCR2, IGSF8, ITPRIPL1, LAG3, PDCD1, PDCD1LG2, SIGLEC15, and TIGIT). The X-axis represents LAMC2 mRNA expression levels (log2[FPKM + 1]). **(K)** Immune checkpoint expression levels in high- and low-LAMC2-expression groups. LAMC2, laminin subunit gamma 2; CD274, programmed cell death 1 ligand 1 (PD-L1); CTLA-4, cytotoxic t-lymphocyte-associated protein 4; HAVCR2, hepatitis A virus cellular receptor 2; IGSF8, immunoglobulin superfamily member 8; ITPRIPL1, inositol 1,4,5-trisphosphate receptor interacting protein like 1; LAG3, lymphocyte-activation gene 3; PDCD1, programmed cell death 1 (PD-1); PDCD1LG2, programmed cell death 1 ligand 2 (PD-L2); SIGLEC15, sialic acid-binding Ig-like lectin 15; TIGIT, T-cell immunoreceptor with Ig and ITIM domains.

This study explored the correlation between LAMC2 mRNA expression and several immune checkpoint molecules. **Figures 2A–J** show the correlation between LAMC2 mRNA expression and immune-related molecules such as CD274 (PD-L1), CTLA-4, HAVCR2, IGSF8, ITPRIPL1, LAG3, PDCD1, PDCD1LG2, SIGLEC15, TIGIT, and others. Notably, CD274, IGSF8, ITPRIPL1, PDCD1LG2, and SIGLEC15 were positively correlated with LAMC2. This suggests that LAMC2 mRNA expression may be closely linked to immune-suppressive molecules (**Figures 2A–J**). Additionally, further analysis dividing samples based on LAMC2 mRNA expression (cutoff = 7.18) revealed that CD274, IGSF8, ITPRIPL1, and SIGLEC15 were significantly elevated in the high-expression group compared to the low-expression group (*P* < 0.05, **Figure 2K**). In contrast, HAVCR2 expression was significantly reduced in the high-expression group (*P* < 0.05, **Figure 2K**).

### LAMC2 high expression drives glycolysis and epithelial–mesenchymal transition pathways, promoting tumor acidic microenvironment formation

**Figure 3:**
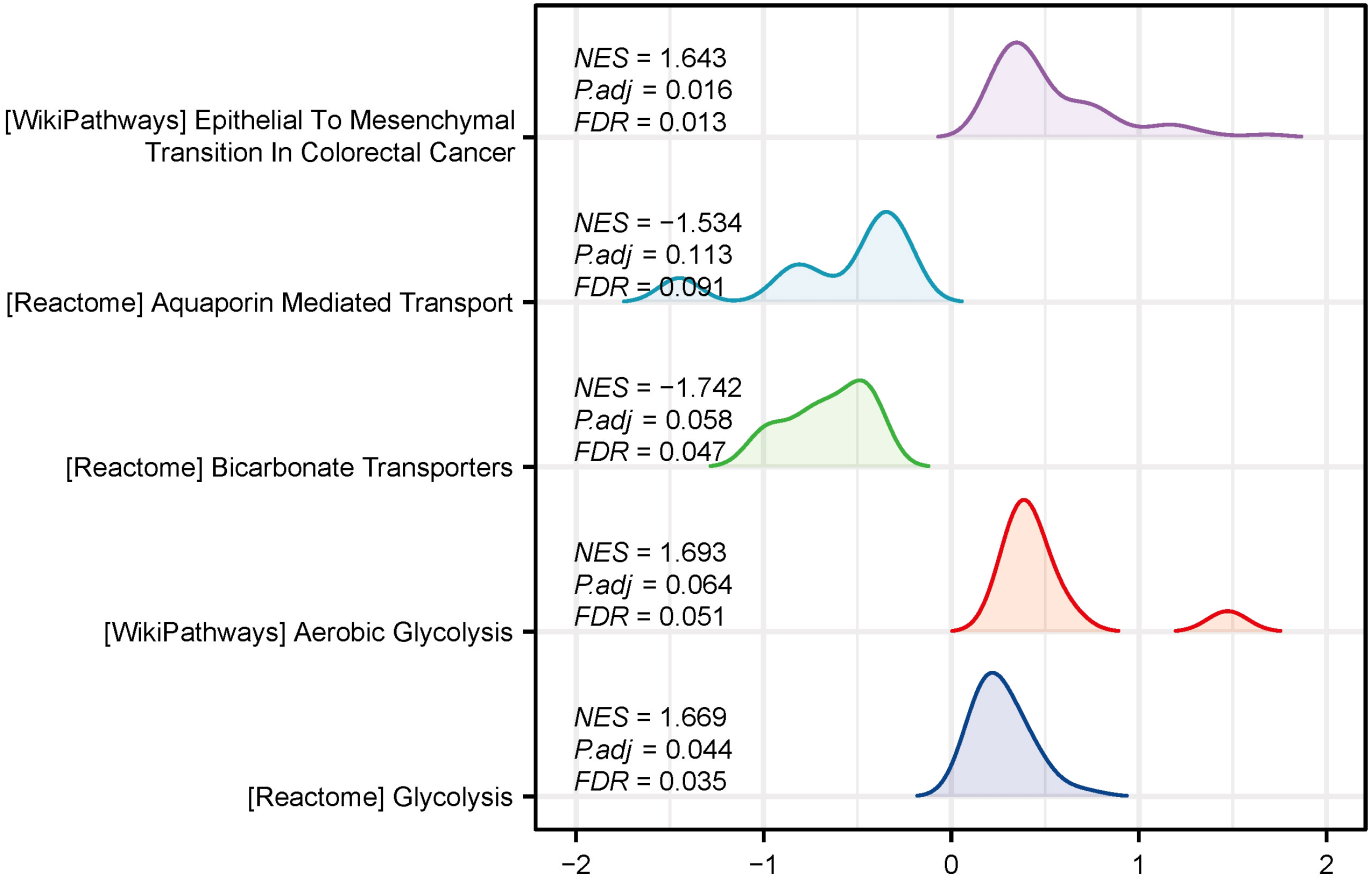
GSEA showing significant changes in glycolysis, epithelial–mesenchymal transition, and aquaporin-mediated transport pathways in the high-LAMC2-expression group.

Gene Set Enrichment Analysis (GSEA) based on LAMC2 expression levels revealed that in the high-expression group, glycolysis pathways (REACTOME_GLYCOLYSIS, NES = 1.669; WP_AEROBIC_GLYCOLYSIS, NES = 1.693) were significantly activated. Conversely, bicarbonate transport (REACTOME_BICARBONATE_TRANSPORTERS, NES = -1.742) and aquaporin-mediated transport (REACTOME_AQUAPORIN_MEDIATED_TRANSPORT, NES = -1.534) functions were impaired, and epithelial–mesenchymal transition (EMT) pathways (WP_EPITHELIAL_TO_MESENCHYMAL_TRANSITION, NES = 1.643) were significantly activated (**Figure 3**). These results suggest that glycolysis-driven lactate accumulation, pH imbalance, and EMT signaling activation together promote the formation of an acidic microenvironment and facilitate tumor malignancy.

### Acidic microenvironment condition screening and LAMC2 expression validation

**Figure 4:**
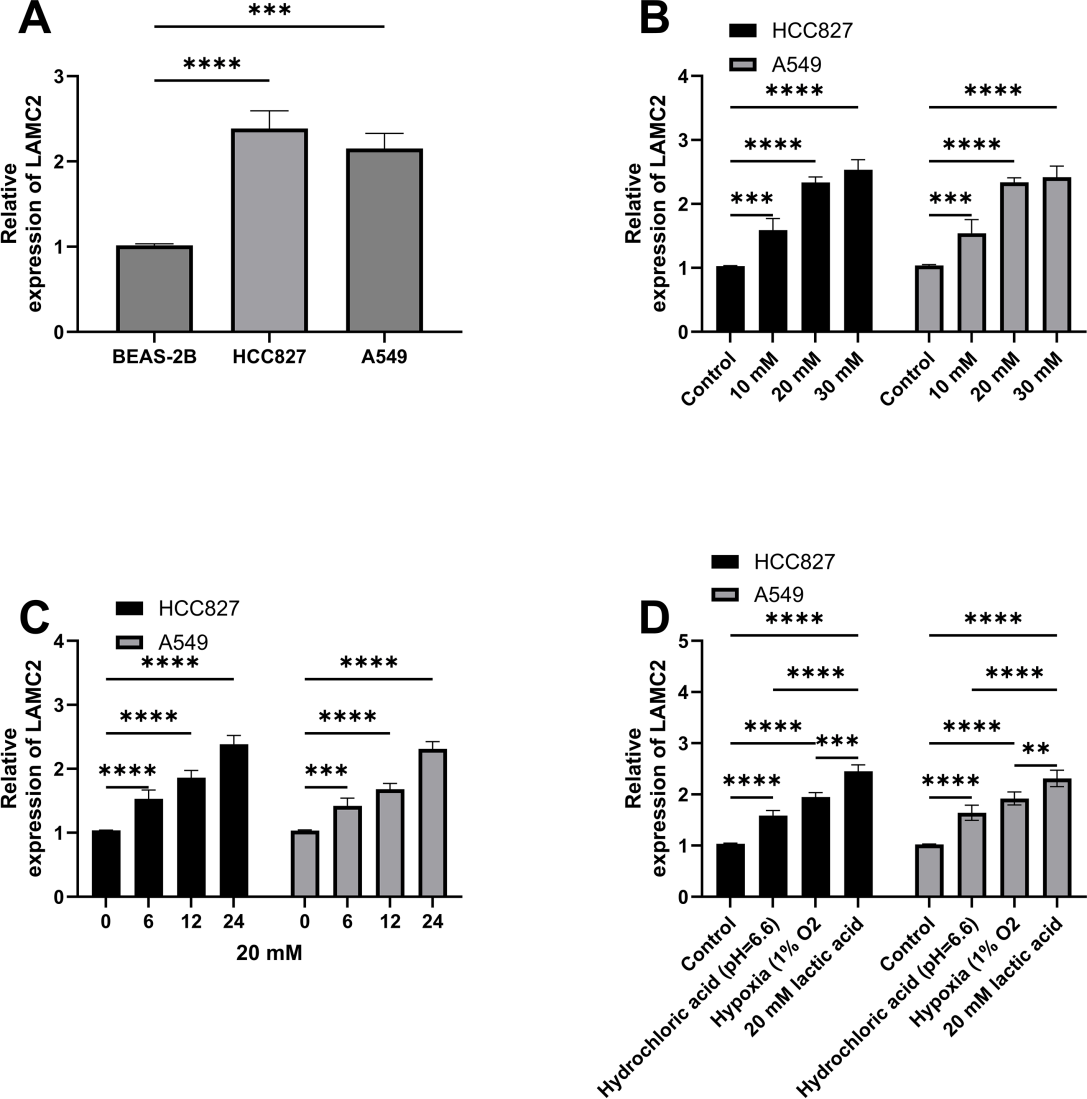
Acidic microenvironment condition screening and LAMC2 expression validation. **(A)** Comparison of relative LAMC2 expression levels in different cell lines (BEAS-2B, HCC827, and A549), showing a significantly higher expression in HCC827 and A549 cells compared to BEAS-2B cells. **(B)** Changes in LAMC2 expression in HCC827 and A549 cells under different lactate **(LA)** concentrations (10, 20, and 30 mM), showing significant increases in LAMC2 expression at 20 and 30 mM concentrations. **(C)** Changes in LAMC2 expression over time (0, 6, 12, and 24 h) in HCC827 and A549 cells treated with 20 mM LA, showing a significant increase in expression with longer treatment times. **(D)** Changes in LAMC2 expression under different acidic environment conditions (high LA, hydrochloric-acid-induced acidosis, and control groups) in HCC827 and A549 cells, with a significantly higher expression in the high LA and hydrochloric-acid-induced acidosis groups. **p < 0.01, ***p < 0.001, ****p < 0.0001.

Initially, LAMC2 expression was measured in lung cancer cells. The qRT-PCR results showed that LAMC2 expression in HCC827 and A549 cells was significantly higher than in BEAS-2B cells (**Figure 4A**). Following treatment with different lactate concentrations, the LAMC2 levels gradually increased in a concentration-dependent manner, with slower increases at 20–30 mM (**Figure 4B**). Additionally, when treated with 20 mM lactate for 24 h, LAMC2 expression gradually increased over time (**Figure 4C**). Moreover, under various acidic microenvironment conditions, particularly with high lactate concentrations, LAMC2 expression was significantly upregulated, suggesting that the acidic microenvironment regulates LAMC2, potentially influencing tumor cell metabolism and aggressive behaviors (**Figure 4D**).

### Effects of lactate treatment on LAMC2, HIF-1α, and c-Myc expression

**Figure 5:**
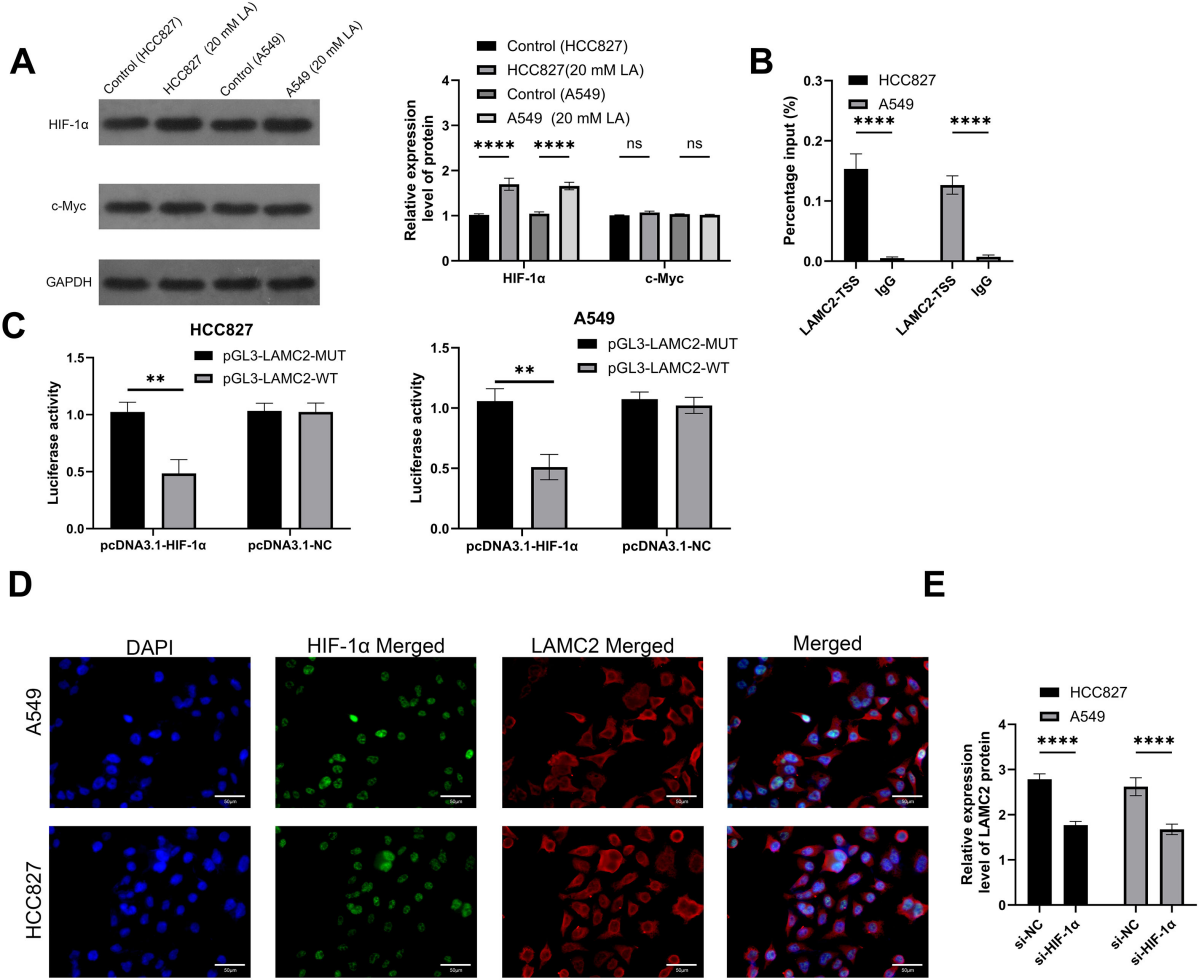
Changes in HIF-1α and LAMC2 expression under lactate treatment. **(A)** Western blot analysis showing the protein expression of HIF-1α in HCC827 and A549 cells treated with lactate (20 mM). **(B)** ChIP analysis showing the increased binding of LAMC2 and HIF-1α in HCC827 and A549 cells under lactate treatment. **(C)** The dual-luciferase reporter assay determined the targeting relationship between HIF-1α and LAMC2. **(D)** Immunofluorescence staining showing the co-expression of HIF-1α (green) and LAMC2 (red) in lactate-treated HCC827 and A549 cells, with DAPI staining for nuclei. **(E)** Significant decrease in LAMC2 expression in HCC827 and A549 cells treated with lactate after HIF-1α knockdown using siRNA. ****p < 0.0001. .

This study analyzed the effects of lactate on LAMC2, HIF-1α, and c-Myc expression. The Western blot results showed that lactate treatment (20 mM) significantly increased HIF-1α protein expression in HCC827 and A549 cells, while c-Myc expression did not significantly change (**Figure 5A**). Chromatin immunoprecipitation (ChIP) analysis revealed that lactate treatment significantly increased the binding of HIF-1α to the LAMC2 promoter in both cell lines (**Figure 5B**). The dual-luciferase reporter assays showed that HIF-1α significantly enhanced the promoter activity of LAMC2 in both HCC827 and A549 cells. The wild-type LAMC2 promoter (pGL3-LAMC2-WT) exhibited a marked increase in luciferase activity after HIF-1α overexpression or lactate treatment, whereas mutation of the HIF-1α binding site (pGL3-LAMC2-MUT) abolished this effect (**Figure 5C**). Immunofluorescence staining (**Figure 5D**) further confirmed that lactate treatment enhanced the co-expression of HIF-1α and LAMC2. Additional experiments validated the regulatory role of HIF-1α on LAMC2 expression (**Figure 5E**).

### Effects of LAMC2 overexpression and knockdown on cell migration, invasion, and proliferation

**Figure 6:**
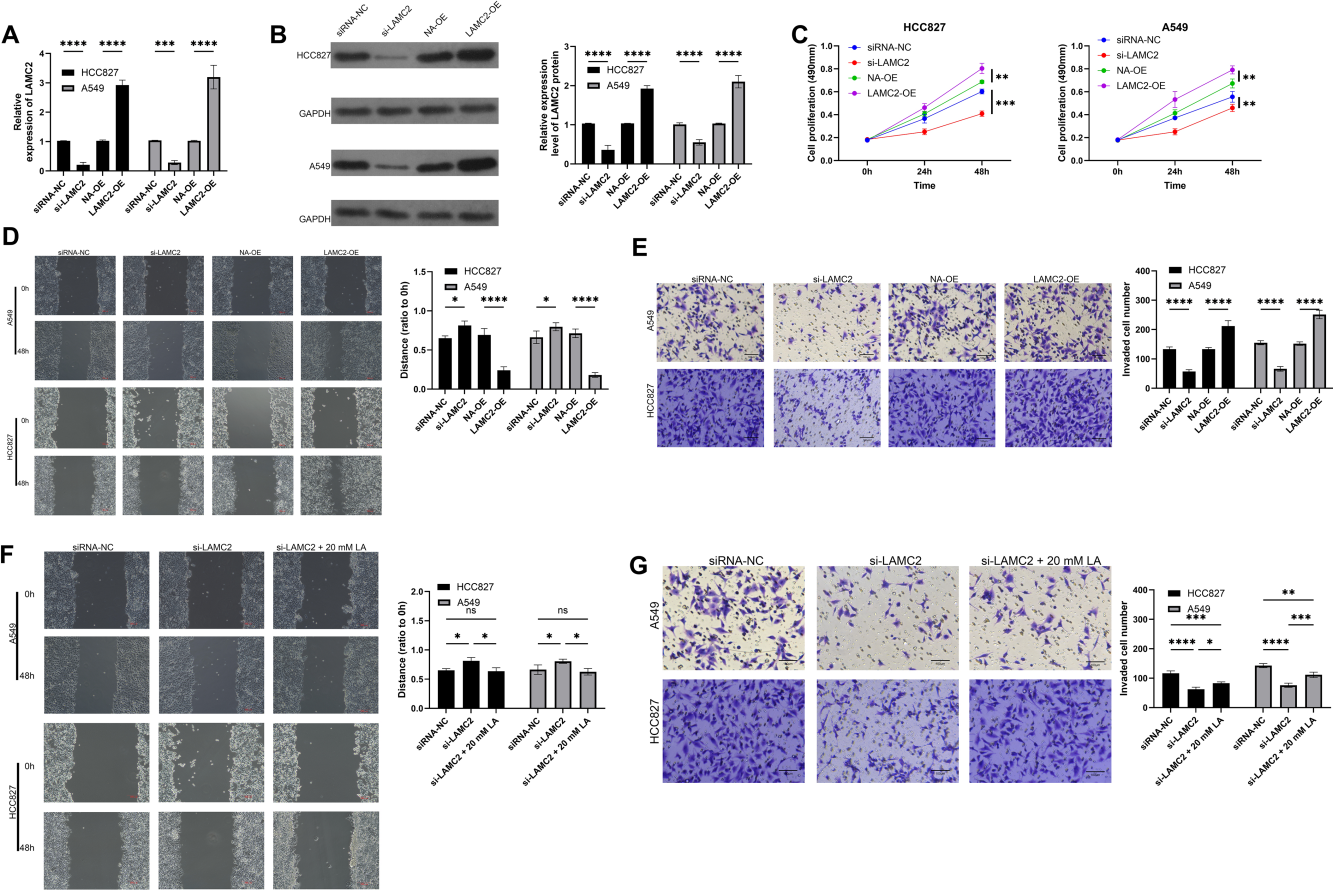
Effects of LAMC2 overexpression and knockdown on cell functions combined with lactate intervention. **(A)** qRT-PCR analysis showing the expression levels of LAMC2 in HCC827 and A549 cells after overexpression (LAMC2-OE) and knockdown (siLAMC2). **(B)** Western blot results showing changes in LAMC2 protein expression in different treatment groups. **(C)** Cell proliferation assay showing the impact of LAMC2 overexpression and knockdown on the proliferation of HCC827 and A549 cells. **(D)** Cell migration assay showing the effects of LAMC2 overexpression and knockdown on the migration ability of HCC827 and A549 cells. **(E)** Cell invasion assay showing the effects of LAMC2 overexpression and knockdown on the invasion ability of HCC827 and A549 cells. **(F)** Cell migration assay under 20 mM lactate intervention, showing the effects of LAMC2 overexpression and knockdown on cell migration. **(G)** Cell invasion assay under 20 mM lactate intervention, showing the effects of LAMC2 overexpression and knockdown on cell invasion. **p < 0.01, ***p < 0.001, ****p < 0.0001.

This study evaluated the effects of LAMC2 overexpression and knockdown on HCC827 and A549 cell migration, invasion, and proliferation. The qRT-PCR analysis confirmed that LAMC2 expression was significantly increased in the overexpression group and decreased in the knockdown group (**Figures 6A,B**). The cell proliferation assays showed that LAMC2 overexpression significantly promoted cell proliferation, while knockdown inhibited it (**Figure 6C**). Migration and invasion assays revealed that LAMC2 overexpression significantly enhanced cell migration and invasion, while knockdown reduced these abilities (**Figures 6D–G**). Moreover, under 20 mM lactate treatment, the migration and invasion capacities of LAMC2-knockdown cells were partially restored compared to their untreated knockdown counterparts, although they still remained lower than those in the lactate-treated control cells (**Figures 6F,G**). These results suggest that LAMC2 plays a critical role in lung cancer cell proliferation, migration, and invasion, and its regulation under lactate conditions further promotes tumor progression.

### LAMC2 regulation of epithelial–mesenchymal transition and signaling pathways in HCC827 and A549 cells

**Figure 7:**
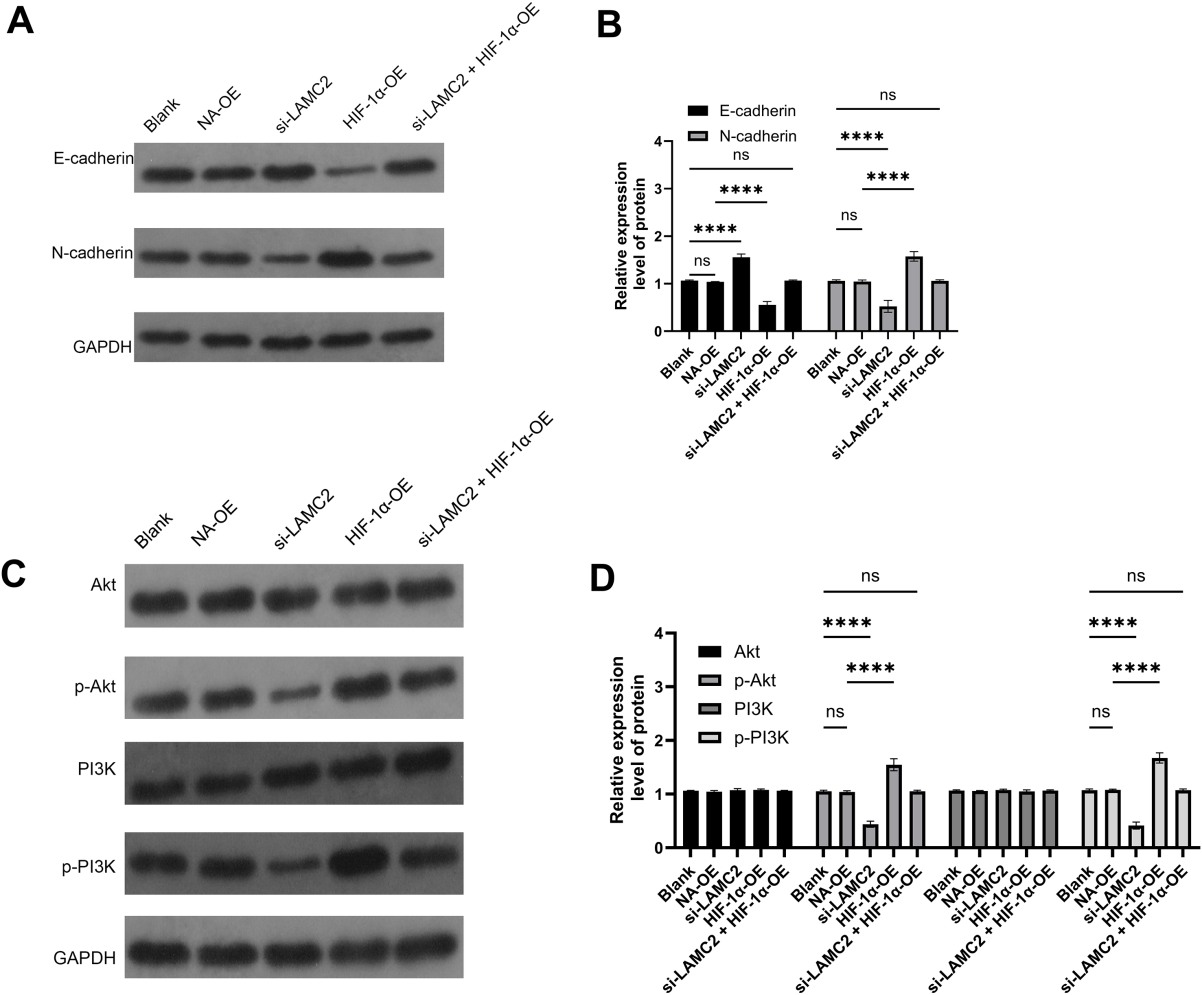
Effect of LAMC2 regulation on epithelial–mesenchymal transition and signaling pathways in HCC827 cells. **(A)** Relative expression levels of E-cadherin and N-cadherin in HCC827 cells (protein bands). **(B)** Expression levels of E-cadherin and N-cadherin in HCC827 cells. **(C)** Relative protein expression levels of Akt, p-Akt, PI3K, and p-PI3K in HCC827 cells (protein bands). **(D)** Expression levels of Akt, p-Akt, PI3K, and p-PI3K in HCC827 cells. ****p < 0.0001.

**Figure 8:**
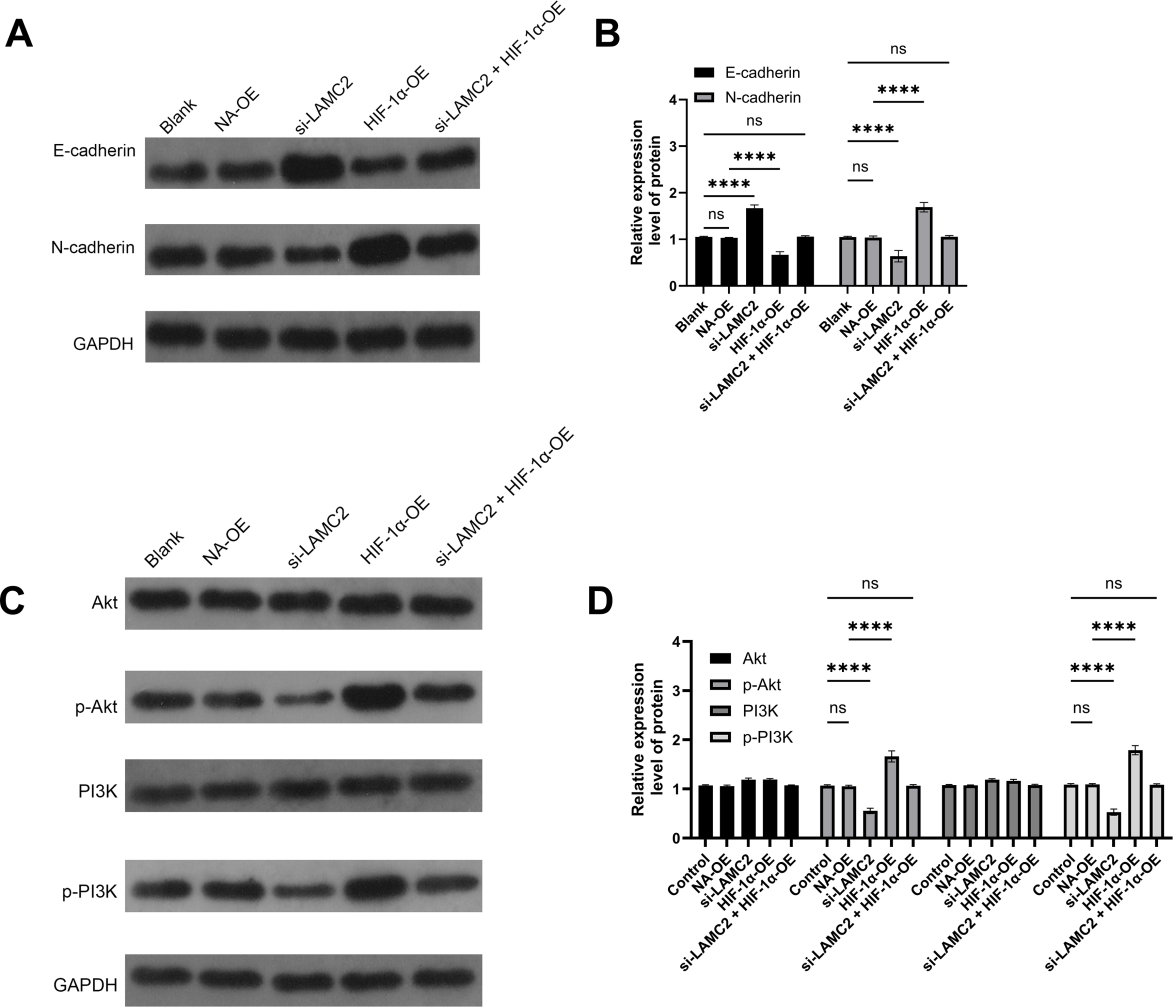
Effect of LAMC2 regulation on epithelial–mesenchymal transition and signaling pathways in A549 cells. **(A)** Relative expression levels of E-cadherin and N-cadherin in A549 cells (protein bands). **(B)** Expression levels of E-cadherin and N-cadherin in A549 cells. **(C)** Relative protein expression levels of Akt, p-Akt, PI3K, and p-PI3K in A549 cells (protein bands). **(D)** Expression levels of Akt, p-Akt, PI3K, and p-PI3K in A549 cells. ****p < 0.0001.

This study assessed the effects of LAMC2 overexpression and knockdown on EMT markers and key signaling pathways in HCC827 and A549 cells. Western blot analysis showed that in HCC827 cells (**Figure 7**) and A549 cells (**Figure 8**), LAMC2 overexpression significantly decreased E-cadherin expression and increased N-cadherin expression, indicating the occurrence of EMT. Similar trends were observed in A549 cells. Further analysis showed that LAMC2 overexpression significantly enhanced the expression of p-Akt and p-PI3K, while total Akt and PI3K protein levels remained unchanged, suggesting that LAMC2 may promote cell growth, migration, and invasion via the PI3K/Akt signaling pathway. In contrast, LAMC2 knockdown significantly decreased the expression of p-Akt and p-PI3K, implying that the PI3K/Akt pathway is inhibited in the absence of LAMC2, which may reduce cell migration and invasion abilities. These findings, in conjunction with the changes in the acidic microenvironment, indicate that the acidic microenvironment may regulate tumor cell biological behavior by modulating LAMC2 expression, further promoting tumor progression.

### LAMC2 expression level and 3-year survival in non-small cell lung cancer patients

**Figure 9:**
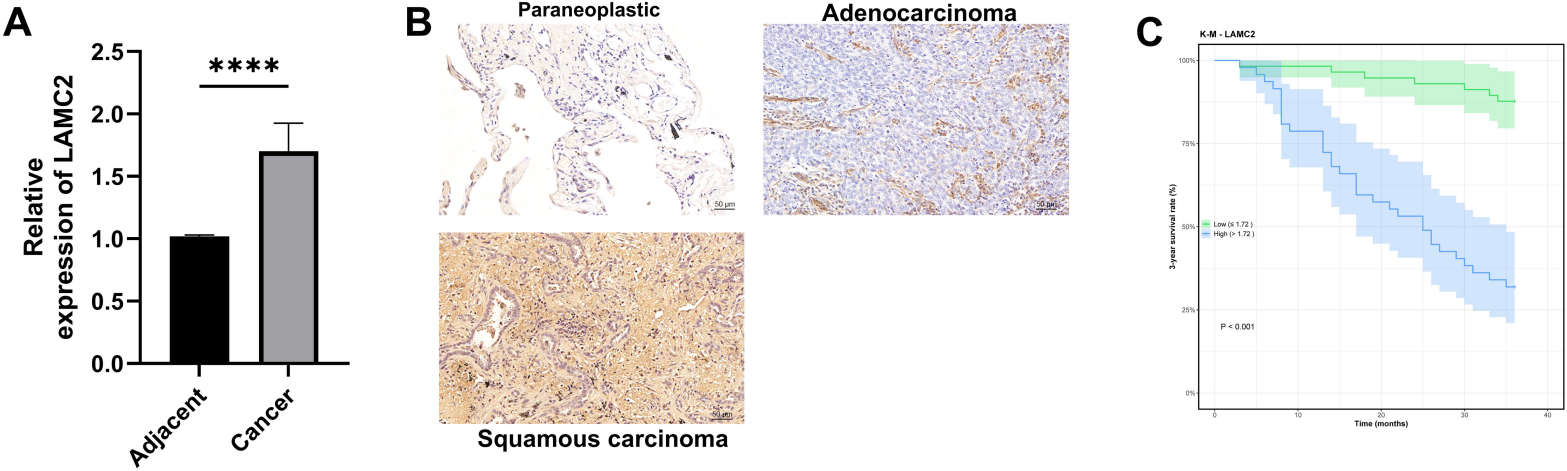
LAMC2 expression in clinical cancer and adjacent normal tissues. **(A)** qRT-PCR analysis showing the relative expression levels of LAMC2 in tumor tissues compared to adjacent normal tissues. **(B)** Immunohistochemical staining results showing LAMC2 expression. **(C)** Three-year survival curve for patients in high- and low-LAMC2-expression groups. ****p < 0.0001.

This study analyzed LAMC2 expression in cancerous and adjacent normal tissues using qRT-PCR and immunohistochemistry (IHC). The qRT-PCR results showed that LAMC2 expression was significantly higher in cancer tissues than in adjacent normal tissues (*P* < 0.0001, **Figure 9A**). The IHC results showed prominent LAMC2 expression in cancer tissues, while expression was lower in adjacent normal tissues (**Figure 9B**). Additionally, they were stratified into high- and low-expression groups based on the optimal cutoff value (1.72) determined from our clinical cohort’s survival data using the surv_cutpoint method. Patients in the high-LAMC2-expression group had a significantly lower 3-year survival rate compared to those in the low-expression group (*P* < 0.001, **Figure 9C**).

### Correlation between LAMC2 expression and patient clinical data

Using the same cutoff (1.72) derived from our cohort’s survival analysis, the patients were divided into high-expression (LAMC2 >1.72) and low-expression (LAMC2 ≤1.72) groups based on the survival analysis cutoff value (1.72), and baseline clinical data were compared. The high-expression group showed significantly higher proportions of poorly differentiated tumors (*P* = 0.010), suggesting that high LAMC2 expression is associated with lower tumor differentiation. Additionally, the high-expression group exhibited significant differences in TNM stage, T stage, and N stage, with more patients in advanced stages (III + IV) (*P* < 0.001), T3 + T4 stage (*P* = 0.014), and N1–3 stage (*P* = 0.006), suggesting that high LAMC2 expression is associated with later stages and greater tumor invasiveness. No significant differences were found for other clinical factors such as age, sex, surgical treatment, radiotherapy/chemotherapy, histological type, or M stage (*P* > 0.05, **Table 1**).

**Table 1:** Correlation between LAMC2 expression and clinical data of non-small cell lung cancer patients.

Factor	Total	High-expression group (*n* = 47)	Low-expression group (*n* = 57)	*χ*^2^-value	*P*-value
Age					
≥60	61 (58.65%)	27 (57.45%)	34 (59.65%)	0.052	0.820
<60	43 (41.35%)	20 (42.55%)	23 (40.35%)		
Gender					
Male	66 (63.46%)	31 (65.96%)	35 (61.40%)	0.230	0.631
Female	38 (36.54%)	16 (34.04%)	22 (38.60%)		
Surgical treatment					
Yes	61 (58.65%)	26 (55.32%)	35 (61.40%)	0.393	0.531
No	43 (41.35%)	21 (44.68%)	22 (38.60%)		
Chemotherapy/radiotherapy					
Yes	43 (41.35%)	21 (44.68%)	22 (38.60%)	0.393	0.531
No	61 (58.65%)	26 (55.32%)	35 (61.40%)		
Histological type					
Squamous ca.	50 (48.08%)	21 (44.68%)	29 (50.88%)	0.396	0.529
Adenoca.	54 (51.92%)	26 (55.32%)	28 (49.12%)		
Degree of differentiation					
Poor	37 (35.58%)	23 (48.94%)	14 (24.56%)	6.677	0.010
Moderate/well	67 (64.42%)	24 (51.06%)	43 (75.44%)		
TNM stage					
I + II	57 (54.81%)	17 (36.17%)	40 (70.18%)	12.026	<0.001
III + IV	47 (45.19%)	30 (63.83%)	17 (29.82%)		
T stage					
T1 + T2	58 (55.77%)	20 (42.55%)	38 (66.67%)	6.072	0.014
T3 + T4	46 (44.23%)	27 (57.45%)	19 (33.33%)		
N stage					
N0	53 (50.96%)	17 (36.17%)	36 (63.16%)	7.507	0.006
N1–3	51 (49.04%)	30 (63.83%)	21 (36.84%)		
M stage					
M0	88 (84.62%)	37 (78.72%)	51 (89.47%)	2.287	0.130
M1	16 (15.38%)	10 (21.28%)	6 (10.53%)		

Squamous ca., squamous carcinoma; Adenoca., adenocarcinoma.

### Cox regression analysis of independent prognostic factors in lung cancer patients

**Figure 10:**
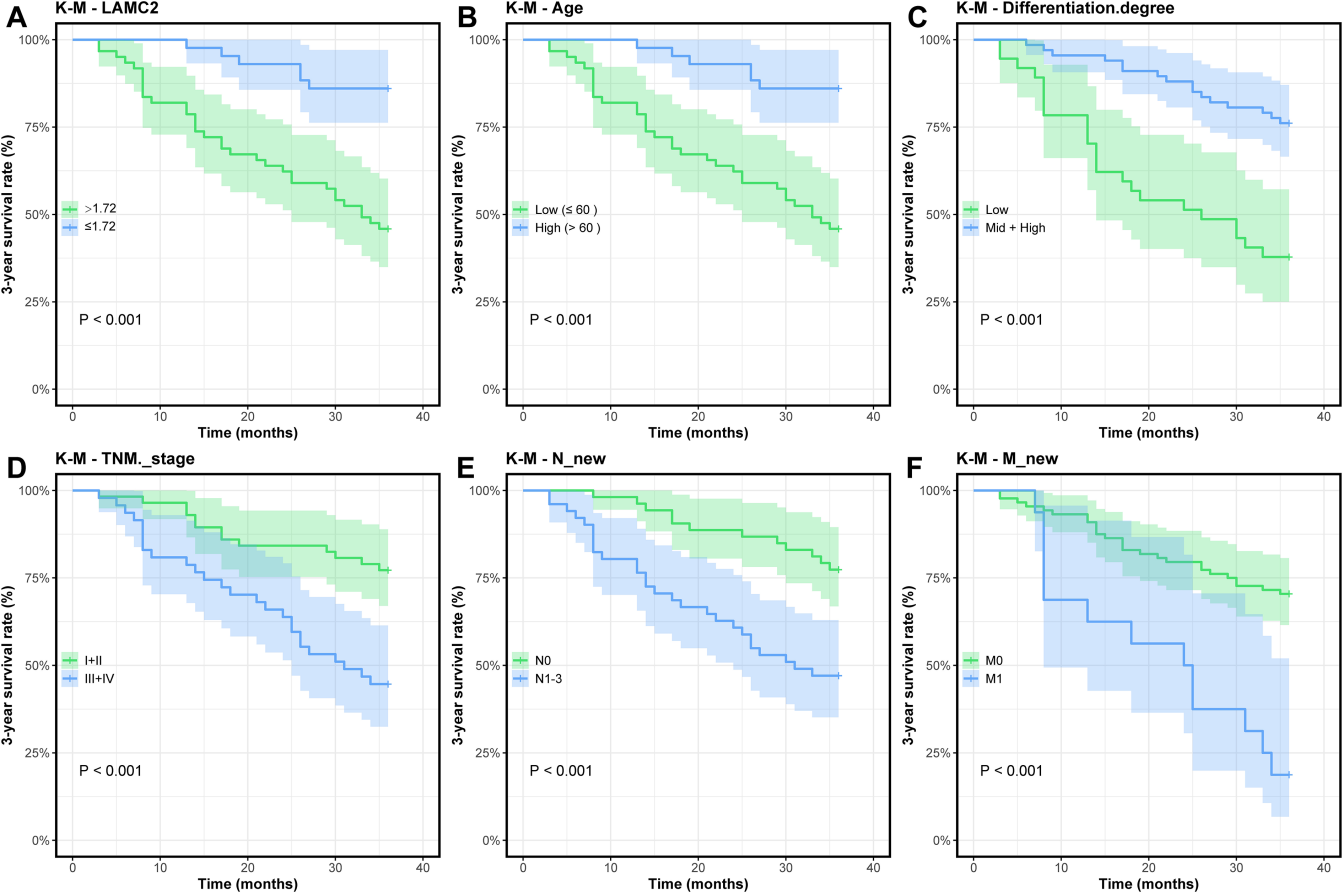
Univariate K–M survival curve: Impact of different variables on lung cancer patient survival. **(A)** Impact of LAMC2 gene expression level on survival prognosis. **(B)** Impact of age on survival prognosis in patients. **(C)** Impact of differentiation level on survival prognosis in patients. **(D)** Impact of TNM stage on survival prognosis in patients. **(E)** Impact of N stage on survival prognosis in patients. **(F)** Impact of M stage on survival prognosis in patients.

Cox regression analysis was used to identify independent prognostic factors for 3-year survival in NSCLC patients. In univariate analysis, low LAMC2 expression (HR = 0.255, 95% CI: 0.129–0.504, *P* < 0.001) and age younger than 60 years (HR = 0.192, 95% CI: 0.080–0.459, *P* < 0.001) were significant predictors of better survival. Well-differentiated patients also exhibited better prognosis (HR = 0.262, 95% CI: 0.138–0.498, *P* < 0.001), while advanced TNM stages (III + IV) (HR = 3.076, 95% CI: 1.578–5.997, *P* < 0.001) and M1 stage (HR = 4.087, 95% CI: 2.086–8.007, *P* < 0.001) were associated with poorer survival (**Figure 10**). No significant effects were observed for other factors such as sex, surgery, radiotherapy/chemotherapy, histological type, or T stage (*P* > 0.05). In multivariate analysis, low LAMC2 expression (HR = 0.207, 95% CI: 0.100–0.428, *P* < 0.001) and age younger than 60 years (HR = 0.149, 95% CI: 0.058–0.383, *P* < 0.001) remained independent prognostic factors. Well-differentiated patients (HR = 0.283, 95% CI: 0.143–0.559, *P* < 0.001) were also significant, while TNM stage, M stage, and N stage did not remain significant (*P* > 0.05) in the multivariate analysis (**Table 2**).

**Table 2:** Cox regression analysis of independent prognostic factors for lung cancer patients.

Factor	*N*	Univariate		Multivariate	
		HR (95% CI)	*P*	HR (95% CI)	*P*
LAMC2					
High	47	Ref.		Ref.	
Low	57	0.26 (0.13–0.50)	<0.001	0.21 (0.10–0.43)	<0.001
Age					
≥60 yr	61	Ref.		Ref.	
<60 yr	43	0.19 (0.08–0.46)	<0.001	0.15 (0.06–0.38)	<0.001
Gender					
Male	66	Ref.		—	—
Female	38	1.13 (0.59–2.15)	0.710		
Surgery					
Yes	61	Ref.		—	—
No	43	1.37 (0.73–2.57)	0.326		
Chemo/RT					
Yes	43	Ref.		—	—
No	61	0.82 (0.44–1.53)	0.530		
Histology					
Squamous	50	Ref.		—	—
Adenocarcinoma	54	0.98 (0.52–1.83)	0.940		
Differentiation					
Poor	37	Ref.		Ref.	
Moderate/high	67	0.26 (0.14–0.50)	<0.001	0.28 (0.14–0.56)	<0.001
TNM stage					
I + II	57	Ref.		Ref.	
III + IV	47	3.08 (1.58–6.00)	<0.001	1.62 (0.50–5.24)	0.417
T stage					
T1 + T2	58	Ref.		—	—
T3 + T4	46	1.43 (0.76–2.68)	0.263		
N stage					
N0	53	Ref.		Ref.	
N1–3	51	3.13 (1.58–6.18)	0.001	2.48 (0.84–7.39)	0.102
M stage					
M0	88	Ref.		Ref.	
M1	16	4.09 (2.09–8.01)	<0.001	1.41 (0.62–3.21)	0.407

HR, hazard ratio; CI, confidence interval; yr, years; Chemo/RT, chemotherapy/radiotherapy; Ref., reference.

## Discussion

**Figure 11:**
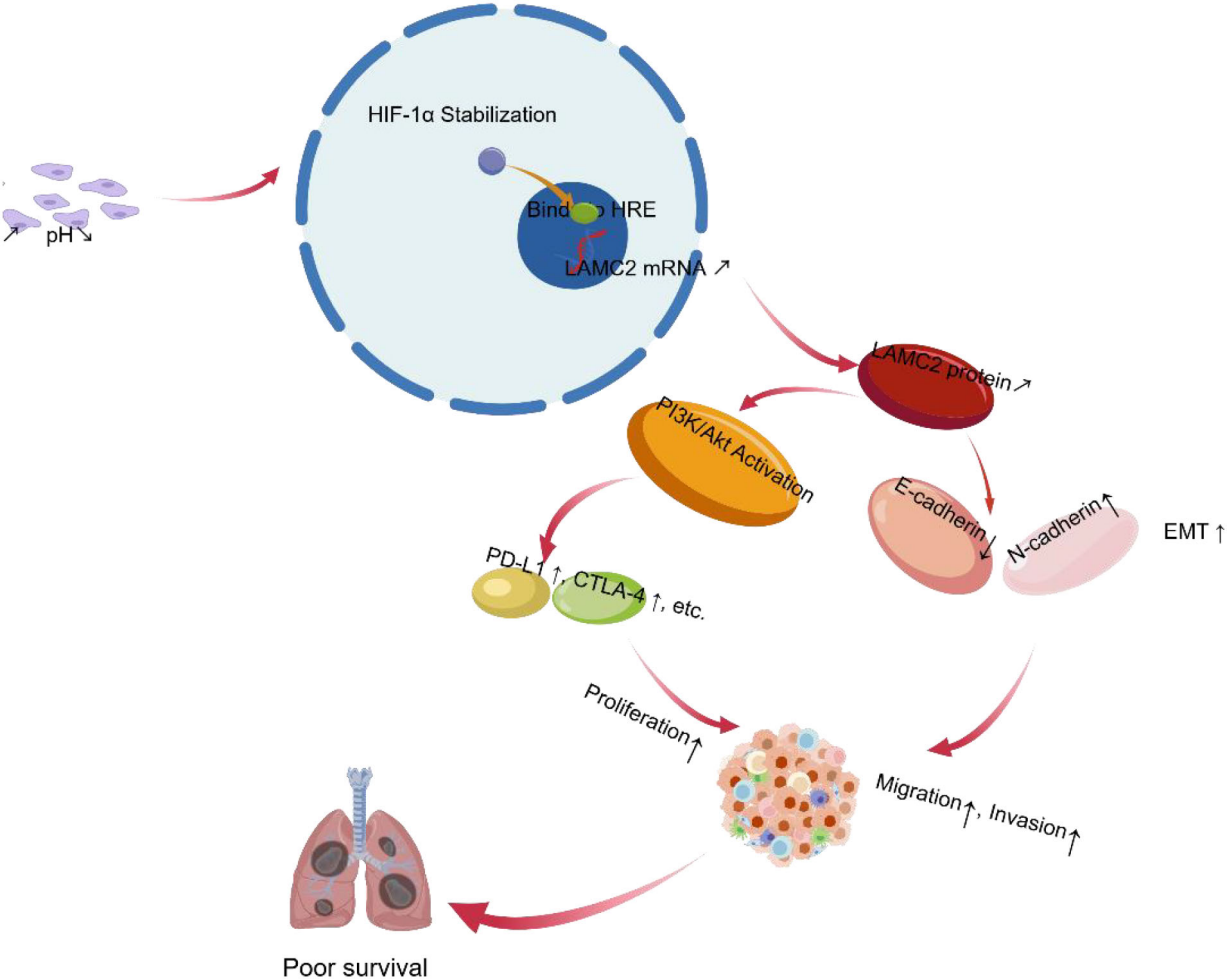
Mechanism of acidic microenvironment-induced LAMC2 expression in NSCLC. The acidic tumor microenvironment (lactate accumulation, low extracellular pH) stabilizes and activates HIF-1α, which translocates to the nucleus and transcriptionally upregulates LAMC2 expression by binding to hypoxia-response elements in its promoter. Elevated LAMC2 activates the PI3K/Akt signaling pathway, promoting cell proliferation and survival and upregulating immune checkpoint molecules (e.g., PD-L1, CTLA-4). LAMC2 also induces epithelial–mesenchymal transition (characterized by decreased E-cadherin and increased N-cadherin), leading to enhanced cell migration and invasion. Clinically, a high LAMC2 expression correlates with poor prognosis and enhanced immune evasion, identifying LAMC2 as a potential therapeutic target for NSCLC.

The acidic microenvironment is a critical characteristic of tumor progression, with lactate accumulation leading to a decrease in local pH (21). The acidic environment promotes tumor cell proliferation and invasion by modulating cell metabolism and gene expression (22). As a key component of the extracellular matrix, LAMC2 may enhance cell adhesion and promote matrix remodeling, thereby driving tumor cell invasiveness (23). This suggests that the acidic microenvironment promotes LAMC2 expression through lactate accumulation, thus exacerbating tumor malignancy. A summary of this proposed mechanism is presented in **Figure 11**.

The glycolytic pathway is the primary energy metabolic pathway for tumor cells in low-oxygen conditions (24). In our study, we found that glycolysis pathways were significantly activated in the high-LAMC2-expression group, which is consistent with previous studies (14). This suggests that LAMC2 may regulate tumor cell metabolism, promote lactate accumulation, and further exacerbate the tumor microenvironment’s acidity. Specifically, many studies have shown (25) that lactate accumulation lowers the local pH, creating an environment favorable for tumor cell proliferation and metastasis. Therefore, the high expression of LAMC2 may promote lactate accumulation in the tumor microenvironment by enhancing glycolysis and accelerating tumor malignancy. From a mechanism perspective, the acidic microenvironment inhibits normal cell functions, increases tumor cell adaptation to hypoxia, and promotes the expression of drug resistance and invasive traits (26). Literature indicates (27) that high LAMC2 expression is closely related to lactate metabolism and may regulate the levels of metabolic products in tumor cells, thus promoting tumor cell invasion and metastasis. Our study also demonstrated that LAMC2 expression is directly related to the activation of glycolysis pathways, further confirming the potential role of LAMC2 in tumor cell metabolic reprogramming.

Additionally, LAMC2 promotes epithelial–mesenchymal transition (EMT), which enhances tumor cell migration and invasion (28). EMT is a key process through which tumor cells acquire migratory and invasive capabilities, and it is closely related to tumor malignant transformation (29). Our study found that LAMC2 regulates the expression of E-cadherin and N-cadherin, promoting the EMT process and further enhancing tumor cell migration and invasiveness. This finding aligns with previous studies (16), suggesting that LAMC2 may promote tumor cell invasiveness not only through extracellular matrix remodeling but also by altering cell adhesion. Furthermore, the PI3K/Akt pathway plays a crucial role in tumor cell proliferation, survival, and migration. We further found that LAMC2 overexpression activated the PI3K/Akt signaling pathway, promoting tumor cell proliferation and migration (30). This mechanism is consistent with existing research (27), indicating that the PI3K/Akt pathway may be the key pathway through which LAMC2 regulates tumor cell invasiveness. Notably, the activation of the PI3K/Akt signaling pathway is closely related to tumor cell metabolic reprogramming, the EMT process, and treatment resistance, which may further exacerbate malignant tumor transformation.

In clinical data analysis, we found that high LAMC2 expression was significantly associated with poor prognosis in NSCLC patients, especially in those with poorly differentiated tumors and advanced TNM stage (31). These findings are consistent with existing literature reports and further validate the potential of LAMC2 as an independent prognostic factor. Literature suggests that high LAMC2 expression is associated with shortened survival, metastasis, and chemoresistance, and its positive correlation with lactate metabolism and tumor cell invasiveness may make it a key regulator in tumor immune escape mechanisms (14). Okada et al. (14) proposed that LAMC2 might promote tumor cell invasiveness and metastatic capacity by regulating lactate accumulation and metabolic reprogramming. Further analysis indicates that high LAMC2 expression may enhance tumor cell adaptation to the acidic microenvironment, further promoting invasiveness and metastasis. Related studies also suggest that LAMC2 may inhibit normal cell function and promote tumor cell adaptive evolution by facilitating lactate accumulation in the tumor microenvironment (32). This finding suggests that LAMC2 is not only a marker of tumor invasion but may also play an important role in tumor immune escape, helping tumor cells survive under harsh microenvironmental conditions, resist treatment, and exacerbate disease progression.

HIF-1α, a key transcription factor activated in hypoxia, regulates various tumor cell behaviors, including lactate metabolism (33). In our study, we found that lactate significantly upregulated LAMC2 expression by activating the HIF-1α pathway. HIF-1α activation may regulate LAMC2 expression either through direct binding or through other signaling pathways, further promoting tumor progression. Basheeruddin et al. (34) indicated that HIF-1α and lactate form an interactive network in the tumor microenvironment, where lactate regulates tumor cell metabolism, proliferation, and invasiveness by activating the HIF-1α pathway. Our findings align with these reports, suggesting that the co-expression of HIF-1α and LAMC2 may promote tumor cell adaptive evolution in the tumor microenvironment, helping tumor cells survive in hypoxic and acidic conditions. Specifically, in tumor cells facing hypoxic and high-lactate environments, HIF-1α may regulate LAMC2 expression to promote metabolic adaptation and enhance tumor invasiveness and metastasis.

Therefore, our study, along with existing literature, suggests that HIF-1α, lactate, and LAMC2 may form a feedback loop to mutually regulate each other, driving tumor invasion and metastasis. In particular, during the survival of tumor cells in low-oxygen and acidic microenvironments, LAMC2, HIF-1α, and lactate may form a tightly regulated network that further promotes tumor invasion and metastasis. The regulation of LAMC2 expression by HIF-1α may be one of the adaptive mechanisms for tumor cells in hypoxic and acidic microenvironments. Through this mechanism, tumor cells can resist external pressure, survive, and expand in the harsh tumor microenvironment. Thus, future research should focus on the functional mechanisms of this regulatory network and explore its potential as a therapeutic target.

Although this study reveals the potential role of LAMC2 in non-small cell lung cancer, it has some limitations. First, the functional validations were conducted exclusively *in vitro* using cell line models, and the absence of *in vivo* animal experiments limits the translational relevance of our findings regarding tumor growth, metastasis, and therapeutic response within a physiological context. Second, the study primarily focused on non-small cell lung cancer and did not involve other tumor types. Future studies should extend to the expression and mechanisms of LAMC2 in other tumor types. Third, this study relies on TCGA database and *in vitro* experimental data, lacking large-scale clinical trial validation. Future studies should focus on (1) elucidating the role of LAMC2 in other tumor types, particularly through *in vivo* models to validate its functional impact in a more physiologically relevant setting, (2) the development of targeted drugs against LAMC2, and (3) the effects of LAMC2 combined with immune checkpoint inhibitors.

## Conclusion

This study found that the acidic microenvironment significantly upregulates LAMC2 expression through lactate accumulation, promoting the malignant progression of non-small cell lung cancer. High LAMC2 expression is closely associated with tumor cell proliferation, migration, and invasion and is correlated with poor prognosis in patients. Further research suggests that LAMC2 may influence the tumor microenvironment through immune escape mechanisms, making it a potential therapeutic target. Targeting LAMC2 or its associated pathways could provide new directions for lung cancer treatment.

## Data Availability

The original contributions presented in the study are included in the article/supplementary material. Further inquiries can be directed to the corresponding authors.
